# Determining energy expenditure in a large seabird using accelerometry

**DOI:** 10.1242/jeb.246922

**Published:** 2023-12-06

**Authors:** Grace J. Sutton, Lauren P. Angel, John R. Speakman, John P. Y. Arnould

**Affiliations:** ^1^ School of Life and Environmental Sciences, Deakin University, 221 Burwood Hwy, Burwood, VIC 3125, Australia; ^2^ Department of Environment & Genetics, and Research Centre for Future Landscapes, La Trobe University, Bundoora, VIC 3086, Australia; ^3^ Institute of Environmental and Biological Sciences, University of Aberdeen, Aberdeen, AB24 2TZ, UK; ^4^ Center for Energy Metabolism and Reproduction, Shenzhen Institutes of Advance Technology, Chinese Academy of Sciences, Shenzhen 518055, China

**Keywords:** Doubly labelled water, Tri-axial accelerometry, Field metabolic rate, Australasian gannet, *Morus serrator*

## Abstract

The trade off between energy gained and expended is the foundation of understanding how, why and when animals perform any activity. Based on the concept that animal movements have an energetic cost, accelerometry is increasingly being used to estimate energy expenditure. However, validation of accelerometry as an accurate proxy for field metabolic rate in free-ranging species is limited. In the present study, Australasian gannets (*Morus serrator*) from the Pope's Eye colony (38°16′42″S 144°41′48″E), south-eastern Australia, were equipped with GPS and tri-axial accelerometers and dosed with doubly labelled water (DLW) to measure energy expenditure during normal behaviour for 3–5 days. The correlation between daily energy expenditure from the DLW and vectorial dynamic body acceleration (VeDBA) was high for both a simple correlation and activity-specific approaches (*R*^2^=0.75 and 0.80, respectively). Varying degrees of success were observed for estimating at-sea metabolic rate from accelerometry when removing time on land using published energy expenditure constants (*R*^2^=0.02) or activity-specific approaches (*R*^2^=0.42). The predictive capacity of energy expenditure models for total and at-sea periods was improved by the addition of total distance travelled and proportion of the sampling period spent at sea during the night, respectively (*R*^2^=0.61–0.82). These results indicate that accelerometry can be used to estimate daily energy expenditure in free-ranging gannets and its accuracy may depend on the inclusion of movement parameters not detected by accelerometry.

## Teaser

**Summary:** Demonstration of the use of accelerometers to estimate energy expenditure/field metabolic rate and to quantify the daily movement patterns of free-ranging Australasian gannets.

## INTRODUCTION

Energy is the fundamental currency for all life as it fuels biological functioning. As there are limits to the rate at which it can be used, the allocation of energy can have important implications for an individual's fitness (McNamara and Houston, 1996). The rate at which energy is expended and acquired can influence how an individual invests in life history traits such as reproduction, growth and survival (Goldstein, 1988). Measuring energy expenditure, therefore, is of fundamental importance for addressing basic biological and ecological questions.

Because of logistical constraints, direct measurement of energy expenditure in free-ranging animals (i.e. field metabolic rate, FMR) is problematic. To date, the most widely used methods have been the doubly labelled water (DLW) method (Speakman, 1997) and the heart rate (*f*_H_) technique (Butler et al., 2004). In addition to the financial costs restricting sample size, both techniques have limitations. For instance, the DLW method provides only a single estimate value averaged over the duration of the study (limited to 24 h to 28 days because of isotope washout rates) and the *f*_H_ method requires invasive procedures to implant loggers (Butler et al., 2004). Furthermore, it is possible that cardiovascular processes unrelated to energy expenditure may influence the relationship between heart rate and oxygen consumption and, thus, the *f*_H_ method requires additional calibrations prior to using it in field settings (Gleiss et al., 2011; Green, 2011).

Accelerometers measure movement in gravitational force of up to three dimensional axes over various temporal scales (Wilson et al., 2006; Brown et al., 2013). Increasingly, accelerometry is becoming a popular method of estimating energy expenditure and is based on the concept that energy must be expended to achieve most movements (Wilson et al., 2020). Overall dynamic body acceleration (ODBA) can be used as a proxy for energy expenditure, obtained from the absolute values of dynamic acceleration (total acceleration minus static acceleration) summed for all three axes (Wilson et al., 2020; Shepard et al., 2008). The accelerometer is typically placed on the centre of the animal's torso as movement in the limbs and extremities can still be detected (Wilson et al., 2006). However, in cases where the device cannot be consistently aligned centrally on the animal's torso, vectorial dynamic body acceleration (VeDBA) is a better proxy for energy expenditure (Gleiss et al., 2011; Qasem et al., 2012), calculated as the square root of the dynamic acceleration summed for all three axes.

A strong correlation between ODBA*/*VeDBA and oxygen consumption or *f*_H_ has been demonstrated for a range of species (mammals, birds, sea turtles) under laboratory conditions (Halsey et al., 2007, 2009, 2011; Wilson et al., 2006; Fahlman et al., 2008; Green et al., 2009), indicating accelerometry alone can be a good proxy for metabolic rate. With the increasing miniaturisation and improved battery life of data loggers (Rutz and Hays, 2009), accelerometry has the potential to enable the collection of behavioural energetics data in free-ranging animals over biologically meaningful durations (Ropert-Coudert and Wilson, 2005; Brown et al., 2012). Correspondingly, an increasing number of studies comparing the relationship between energy expenditure (measured using DLW or *f*_H_) and accelerometry have been conducted on free-ranging species (Sutton et al., 2021; Elliott et al., 2013; Hicks et al., 2020, 2017; Pagano and Williams, 2019). Within avian studies, these relationships have been determined largely in smaller, aquatic species with a high proportion of foraging time spent submerged (Elliott et al., 2013; Ste-Marie et al., 2022; Sutton et al., 2021). Relatively few studies have investigated these relationships in larger volant species (Stothart et al., 2016; Brown et al., 2022).

Throughout the world, seabirds are major consumers of marine biomass and play an important role in determining ecosystem structure and function (Boyd et al., 2006; Furness and Monaghan, 1987). Anticipated fluctuations in ocean currents and environmental variability due to climate change are likely to have major impacts on the distribution and abundance of seabird prey in many parts of the world (Barbraud et al., 2012). Knowledge of how seabirds apportion energy to various behaviours, and the factors affecting this, is crucial to understanding how they may respond to such changes. Consequently, there is a need for developing and validating widely applicable techniques capable of determining activity-specific energy expenditure in these species (Elliott et al., 2013).

The Australasian gannet (*Morus serrator*), is a large, wide-ranging pelagic seabird (Nelson, 1978) and an ideal candidate for determining activity-specific energy expenditure because of the limited suite of behaviours which are reflected in distinct accelerometry signals (Ropert-Coudert et al., 2004a). Furthermore, while gannets display high-energy plunge diving, this behaviour represents only a small proportion of their at-sea activities, which include gliding and flapping flight (Angel et al., 2015). The aims of the present study, therefore, were to determine whether tri-axial accelerometry can be used to: (1) accurately predict metabolic rate; and (2) develop activity-specific energy estimates in the Australasian gannet. The relationship was investigated over two temporal periods, namely the entire sampling period and the at-sea duration of the sampling period.

## MATERIALS AND METHODS

### Study site and animal handling

The study was conducted on 4–10 December 2014 at the Pope's Eye Australasian gannet colony (38°16′42″S, 144°41′48″E) in south-eastern Australia. Adult *Morus serrator* (Gray, G. R. 1843) raising young, downy chicks (20–50 days old; Wingham, 1984) were randomly selected throughout the colony. Adults were captured by hand at the nest and the chick was covered for protection from conspecifics during procedures. All animal handling followed protocols approved by Deakin University Animal Welfare Committee [A86/2010] and the Department of Sustainability and Environment Victoria Wildlife Research [Permit 0005745]. Individuals were weighed in a cloth bag by suspension scale (±25 g, Salter Australia Pty Ltd) before exposed culmen (bill length), bill depth and tarsus length were measured using Vernier callipers (±0.1 mm), and total head length was measured using a slide ruler (±1 mm). Wing chord length could not be measured because of feather deterioration potentially biasing results (Coulson, 2009) and, hence, the length of the ulna bone (hereafter referred to as the wing ulna) was measured with a slide ruler (Rising and Somers, 1989).

To determine the at-sea movements of breeding birds, individuals were equipped with a GPS data logger (IgotU GT-600, Mobile Action Technologies Inc., New Taipei City, Taiwan; 26.5 g) recording location (±10 m) every 2 min. To obtain information on activity patterns and foraging behaviour (Shepard et al., 2010), all individuals were also instrumented with a tri-axial accelerometer data logger (X8, Gulf Coast Data Concepts LLC, Waveland, MS, USA; 14.12 g) sampling at 25 Hz. Before deployment of loggers, a bench test was performed to ensure they were functional and recording on the correct axes within similar ranges (further information regarding devices and manufacturer testing are available from figshare: doi:10.6084/m9.figshare.24418762). The devices were encapsulated in heat shrink plastic (whole package 52.6 g; <3% body mass) and attached with water-proof tape (Tesa^®^ 4651, Beiersdorf AG, Hamburg, Germany) to the central tail feathers following the methods of Wilson et al. (1997). Device positioning ensured it was covered by the wings during a plunge dive in an attempt to reduce initial impact and hydrodynamic drag (Hamer et al., 2000).

An initial blood sample (0.1 ml) to establish background levels of ^2^H and ^18^O (method D of Speakman and Racey, 1987) was collected by venipuncture of a tarsus vein before individuals were administered an intraperitoneal injection (1.85±0.03 ml) of DLW (64.3% ^18^O and 34.1% ^2^H). Syringes were weighed before and after injection to calculate the mass of DLW injected into each bird (±0.001 g, FX300i milligram balance, A&D Company Ltd). Birds were returned to their nest for 2–6 h (3.36±0.09 h), allowing for equilibration of the isotopes with the body water pool, before a second blood sample was collected. Individuals were then left to resume normal behaviour, including foraging at sea, for 3–5 days before being recaptured at the colony, weighed, their devices removed, and a final blood sample collected. Whole-blood samples were stored in flame-sealed glass capillary tubes at room temperature until isotopic analysis 2 months later.

### Data processing and statistical analyses

Data obtained from the tri-axial accelerometers were used to visually assess behaviour in IGOR Pro (version 6.34, WaveMetrics) based on previous studies of plunge-diving species (Ropert-Coudert et al., 2004b, 2009; Weimerskirch et al., 2005). Five key behaviours were identified: flapping flight, gliding flight, resting on the sea surface, resting on land, and foraging (i.e. diving). The *Ethographer* package was used to perform a *k*-means algorithm clustering analysis (see Sakamoto et al., 2009) and identify behaviours using an unsupervised continuous wavelet transformation (1 s window). Each cluster was assigned a behaviour based on the previous identification. Additional conditions were applied in the R statistical environment (http://www.R-project.org/) to identify miss-classification of behaviours produced by similar movement. For example, travelling speed was used to separate gliding from resting on the sea surface, while GPS location was used to discern resting on land from resting at sea. Additionally, gannets perform both plunge dives and duck dives (Warwick-Evans et al., 2015; Machovsky-Capuska et al., 2011). Dive type was determined by whether a gannet was in flight prior to a dive (plunge dive) or resting on the sea surface (duck dive). The time spent performing each behaviour during the whole study period was then calculated to obtain a single value per individual.

All following statistical analyses were performed using the R statistical environment (http://www.R-project.org/). The downloaded GPS locations were processed using a speed filter (McConnell et al., 1992) and summary statistics were calculated using the *adehabitatHR* package (Calenge, 2006). Trip parameters indicative of foraging effort (total distance travelled and trip duration) were calculated for each foraging trip and summed for the duration of the study (i.e. from the time of the equilibration blood sample to the final blood sample).

From the raw tri-axial data, the static (reflecting animal positioning with respect to gravity) and dynamic acceleration were separated (Shepard et al., 2008). The dynamic acceleration for each axis, *X* (surge), *Y* (sway) and *Z* (heave), was then used to calculate dynamic body acceleration. As devices placed on the back of the bird have a high likelihood of being dislodged during the high-speed dives of gannets, the accelerometer could not be placed in the animal's centre of gravity. Thus, VeDBA was used as an estimate of energy expenditure (Qasem et al., 2012; Gleiss et al., 2011). VeDBA (***g***) was calculated as:
(1)

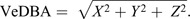
The sum and mean (VeDBA_sum_, VeDBA_mean_, respectively) were calculated for the total sampling period (VeDBA_sum-T_, VeDBA_mean-T_) as well as the at-sea sampling period (VeDBA_sum-S_, VeDBA_mean-S_).

Isotope enrichment of blood samples was determined by isotope ratio mass spectrometry (Optima IRMS and Isochrom μG, Micromass, Manchester, UK). Specifically, ^2^H enrichment was determined by online chromium reduction and ^18^O enrichment was determined from the small-sample equilibration technique (Speakman and Król, 2005). The injectate enrichment was estimated from the average of five subsample solutions (consisting of the original injectate diluted with tap water), processed through mass spectrometry (Speakman, 1997). The initial total body water pool was determined from the ^18^O dilution space using the plateau method (Speakman, 1997). Final total body water pool was calculated from body mass, assuming the pool contributed a constant proportion of the total mass of the animal throughout the study (Lifson and McClintock, 1966). Isotope enrichment levels were converted into total energy expenditure (DLW_EE_; kJ) based on the single pool model and assuming 25% evaporative water loss (see equation 7.17 of Speakman, 1997). Daily energy expenditure (DLW_DEE_; kJ day^−1^) for each individual was calculated by dividing DLW_EE_ by the duration between the bird's first and last blood sample.

To assess whether accelerometry on its own can reliably predict daily energy expenditure in breeding gannets, the relationship between DLW_DEE_ and VeDBA_mean_ was determined for the total sampling period modelled using a linear regression. The coefficient of determination (*R*^2^) was used to assess the strength of the relationship between DLW_DEE_ and VeDBA_mean_. As this direct correlative approach does not always reveal a relationship (Elliott et al., 2013; Jeanniard-du-Dot et al., 2017a), energy expenditure from VeDBA was also determined through an activity-specific approach. As outlined in Jeanniard-du-Dot et al. (2017a), VeDBA values were summed within each behaviour identified through *k*-means clustering analysis to determine activity-specific energy expenditure. To estimate activity-specific energy expenditure, a linear regression was fitted using the activity-specific VeDBA_sum_ values for each individual (DLW_EE_=*T*_behaviour(i)_+*T*_behaviour(ii)_…, where *T* is time). The parameter estimates were then added into the following equation:
(2)

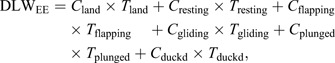
where *C_i_* is the parameter estimate for the rate of energy expenditure (kJ) for each activity and *T_i_* is the time spent per activity (h) as determined from the accelerometry data, for the key activities resting on land (land), resting at sea (resting), flapping flight (flapping), gliding flight (gliding), plunge diving (plunged) and duck diving (duckd). The coefficients were used to predict total energy expenditure (Pred_EE_, kJ), which was converted to an estimate of predicted metabolic rate (Pred_DEE_, kJ day^−1^).

On-land energy expenditure could not be determined by the DLW method as there were no individuals that remained on land for the entire sampling period. Consequently, it was not possible to calculate at-sea energy expenditure in individuals by deducting DLW estimates of on-land energy expenditure from the total energy expenditure for individuals that went to sea, as has been done in previous studies (Sutton et al., 2021). Instead, two alternative approaches were investigated for determining on-land energy expenditure to then be deducted from the total sampling period. In the first approach (DLW_EE-S1_, kJ), the activity-specific VeDBA values were fitted to the energy expenditure estimates derived from Eqn 2 (i.e. *C*_behaviour_×*T*_behaviour_) for land periods and subtracted from the total energy expenditure measured through the DLW method:
(3)

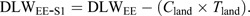
In the second approach (DLW_EE-S2_, kJ), published estimates of on-land metabolic rates were subtracted from the measured total energy expenditure. As *f*_H_, and consequently energy expenditure, has been shown to vary between day and night (Green et al., 2013), time spent on the nest was apportioned into night and day periods at Pope's Eye (https://www.timeanddate.com/sun/). The energy expended on land (DLW_EE-land_, kJ) was then calculated by the following equation:
(4)

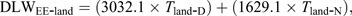
where constants were derived from Green et al. (2013) and multiplied by the time spent on land during the day and the night (*T*_land-D_, *T*_land-N_, respectively). At-sea energy expenditure (kJ) was then calculated using the following equation:
(5)

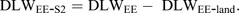
The daily rates of energy expended at sea (DLW_DEE-S1_ and DLW_DEE-S2_, kJ day^−1^) were then calculated by dividing the values derived from Eqn 3 and Eqn 5 by the time spent at sea during the study period. The relationships between DLW_DEE-S_ and VeDBA_mean-S_ were modelled and the coefficient of determination was used to assess the strength of the relationship.

To establish whether relationships between energy expenditure derived from DLW and accelerometry could be improved, linear models were used to incorporate parameters likely to influence energy expenditure (i.e. total distance travelled, dive rate, body mass, proportion of sampled period spent at sea, and tarsus length as an indication of size). For the at-sea models, the proportion of time spent at sea during the night was included instead of the proportion of the sampled period spent at sea. Model selection was conducted using stepwise (forward and backward) regression using the *MASS* package (https://CRAN.R-project.org/package=MASS). All results are reported as means±s.e.m. unless otherwise stated. Data used in this study are available in Table S1.

## RESULTS

Accelerometer, GPS and DLW data were obtained from a total of 21 birds. However, six loggers failed while individuals were at sea, resulting in 15 complete sets of DLW and movement data (9 males, 6 females; Table 1). Individuals were sampled for 74.0±5.2 h (range: 21.3–114.9 h) where they expended 9995±1194 kJ at a rate of 3172±232 kJ day^−1^ (Table 2). There were no statistically significant differences between body mass measured at the beginning (2.61±0.05) and the end (2.67±0.04) of the instrumentation period (*t*_14_=1.16, *P=*0.13). Comparison between the sexes revealed no statistically significant differences in body mass (Kruskal–Wallis: *H*_1_*=*0.58, *P=*0.44), foraging trip distance (*H*_1_*=*0.06, *P=*0.81), duration (*H*_1_*=*0.01, *P=*0.90) and DLW_DEE_ (*H*_1_*=*0.34, *P=*0.56), and thus data were combined.

**Table 1.**
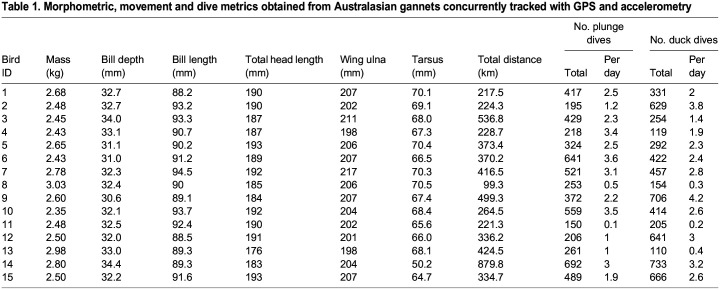
Morphometric, movement and dive metrics obtained from Australasian gannets concurrently tracked with GPS and accelerometry

**Table 2.**
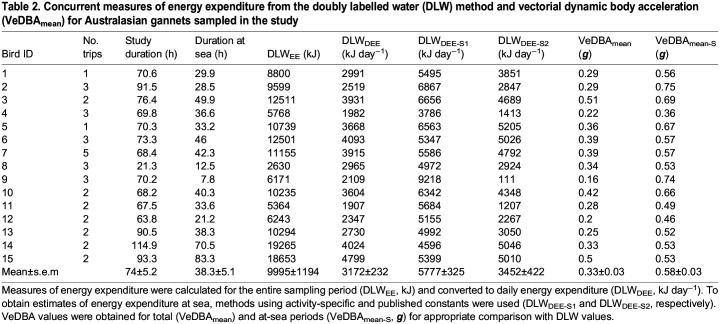
Concurrent measures of energy expenditure from the doubly labelled water (DLW) method and vectorial dynamic body acceleration (VeDBA_mean_) for Australasian gannets sampled in the study

**Fig. 1.**
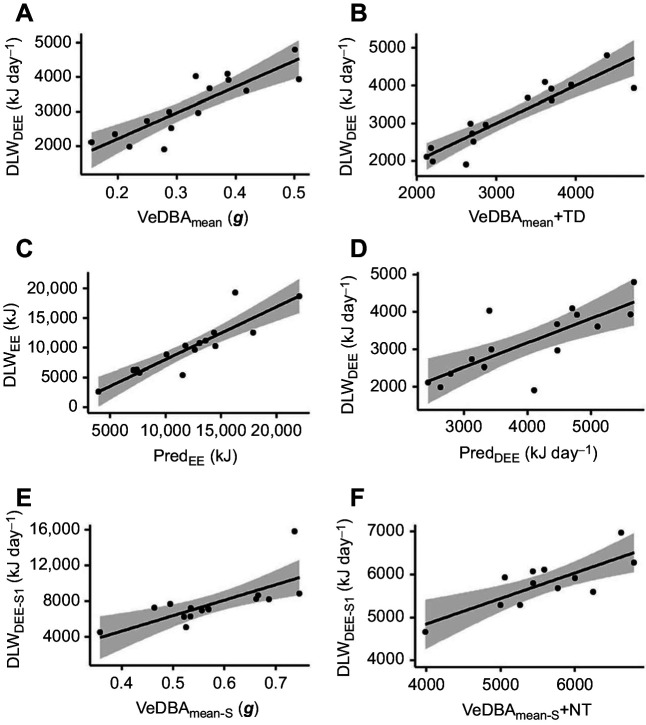
**Relationships between energy expenditure calculated using the doubly labelled water method and accelerometers.** Correlations between estimated energy expenditure derived from the daily, total or at-sea energy expenditure (DLW_DEE_, DLW_EE_ or DLW_DEE-S1_, respectively) and: mean vectorial dynamic body acceleration (VeDBA_mean_; A, *R*^2^=0.75); and activity-specific energy expenditure as predicted through multiple regression incorporating activity budgets presented as total and daily rates (Pred_EE_ and Pred_DEE_, respectively; C, *R*^2^=0.80; and D, *R*^2^=0.60). Removal of time on land revealed correlations between daily energy expended at sea (DLW_DEE-S1_) and mean VeDBA at sea (VeDBA_mean-S_; E, *R*^2^=0.42). The most parsimonious models for predicting DLW_DEE_ and DLW_DEE-S1_ incorporated the predictor variables total distance travelled (TD) and proportion of time spent at sea at night (NT) (models B and F, respectively; B, *R*^2^=0.82; and F, *R*^2^=0.69). Plots show the linear regression (solid line) and 95% confidence interval (shaded).

**Fig. 2.**
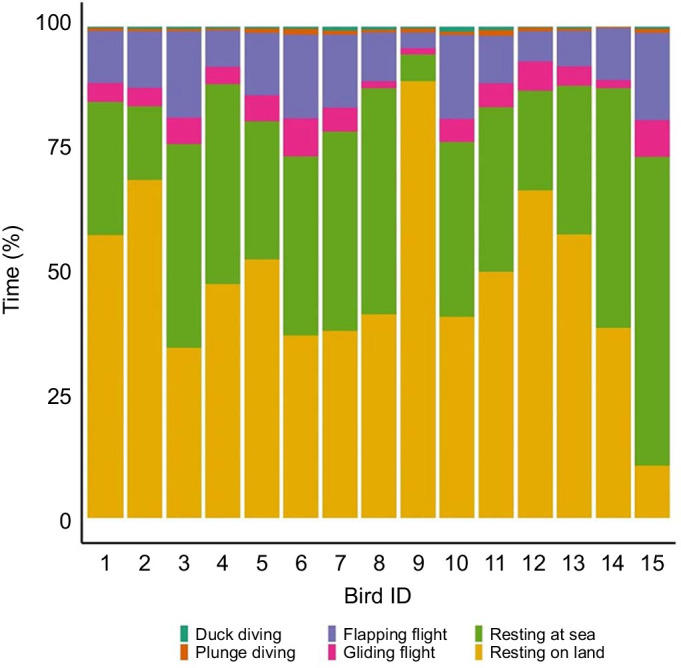
**Variation between individuals in the proportion of time spent performing the different behaviours.** Five key behaviours were identified: duck diving (dark green), plunge diving (brown), flapping flight (purple), gliding flight (pink), resting at sea (light green) and resting on land (yellow).

VeDBA_mean_ for each individual across their total sampling period was 0.33±0.03 g. It was found to be positively correlated with the daily rate of energy expenditure (DLW_DEE_, *R*^2^=0.75, *F*_1,13_=38.4, *P*<0.001; Fig. 1A, Table 3), giving the relationship:
(6)

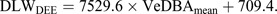
The most parsimonious model for DLW_DEE_ included total distance travelled (TD) and VeDBA_mean_ (Table 3). The inclusion of this parameter increased the predictive strength of the relationship (*R*^2^=0.80, *F*_1,13_=23.4, *P<*0.001; Fig. 1B):
(7)

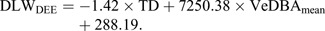
Individuals spent 47.6±3.9% of the study period (35.7±4.1 h) at the colony (Fig. 2). On average, 38.3±5.1 h (range: 7.8–83.3 h) was spent at sea, during which time 1–5 foraging trips were made, covering a total distance of 361.8±47.9 km (Table 1; range: 99.3–879.8 km). At sea, the highest proportion of time was spent resting (34.7±3.6%), followed by time spent in flight (11.7±1.0% flapping flight, 4.6±0.4% gliding flight). Diving behaviours comprised only 2.7±0.5% of the study period, with individuals completing a total of 381±44 plunge dives (1.7±0.4%) and 408±57 duck dives (1.0±0.1%) at a rate of 2.1±0.2 and 2.2±0.3 dives h^−1^, respectively (Table 1).

**Table 3.**
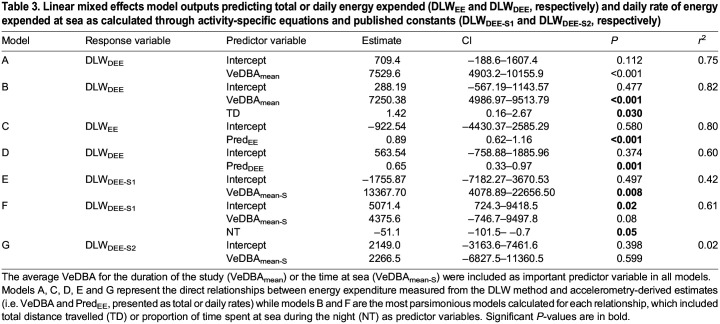
Linear mixed effects model outputs predicting total or daily energy expended (DLW_EE_ and DLW_DEE_, respectively) and daily rate of energy expended at sea as calculated through activity-specific equations and published constants (DLW_DEE-S1_ and DLW_DEE-S2_, respectively)

**Fig. 3.**
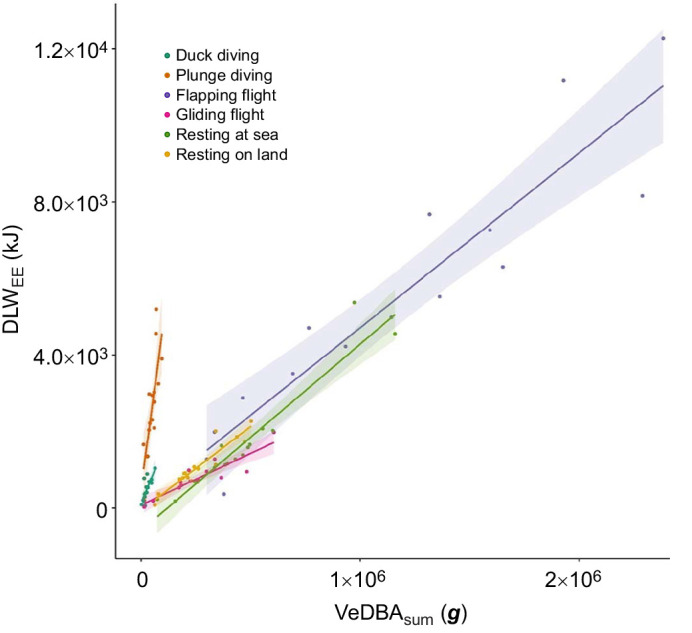
**Estimates of activity-specific energy expenditure calculated from Eqn 2.** Plot shows the predicted model linear regression (solid line) and 95% confidence interval (shaded) for each activity: duck diving (dark green), plunge diving (brown), flapping flight (purple), gliding flight (pink), resting at sea (light green) and resting on land (yellow).

The activity-specific VeDBA values were compared with the activity estimates determined from DLW_EE_ in Eqn 2 to develop energy expenditure estimates for each behaviour (Fig. 3). Strong positive correlations (i.e. *R^2^*≥0.80) were found for at-sea resting (*R*^2^=0.91, *F*_1,13_ =135.8, *P<*0.001), resting on land (*R*^2^=0.88, *F*_1,13_=95.6, *P<*0.001), flapping flight (*R*^2^=0.87, *F*_1,13_=85.5, *P<*0.001) and gliding flight (*R*^2^=0.80, *F*_1,13_=53.3, *P<*0.001; Table 3). Weaker but significant correlations were determined for plunge diving (*R*^2^=0.60, *F*_1,13_=19.53, *P<*0.001) and duck diving (*R*^2^=0.57, *F*_1,13_=17.1, *P<*0.001) behaviours (Table 3).

Modelling of DLW_EE_ and the predicted total energy expenditure (Pred_EE_) of the whole sampling period revealed a strong positive relationship (*R*^2^=0.80, *F*_1,13_=51.2, *P<*0.001; Fig. 1C):
(8)

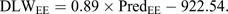
However, estimates which include time on both sides of the equation are known to result in an overestimated relationship (Ladds et al., 2017; Halsey, 2017). Therefore, values were converted to daily energy expenditure (DLW_DEE_, kJ day^−1^) and compared with VeDBA_mean_ (***g***) to assess relationships without the presence of a temporal bias. A significant, albeit slightly weaker, relationship (*R*^2^=0.60, *F*_1,13_=19.4, *P<*0.001; Fig. 1D) was observed:
(9)

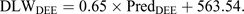
The mean vectorial dynamic body acceleration of the at-sea period (VeDBA_mean-S_) was 0.58±0.03 g (Table 2). As the accelerometry-derived activity-specific relationships with DLW were strong (Table 4), at-sea energy expenditure could be estimated. After subtracting the energy expended relating to land periods from DLW_EE_, at-sea daily energy expenditure (DLW_DEE-S1_) was calculated to be 5931±562 kJ day^−1^. This was found to be positively correlated with VeDBA_mean-S_ (*R*^2^=0.42, *F*_1,13_=9.7, *P<*0.01; Fig. 1E), giving the relationship:
(10)

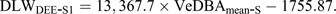
The most parsimonious model for DLW_DEE-S1_ included the proportion of time spent at sea during the night (NT) and VeDBA_mean-S_ (Table 3). The inclusion of this parameter strengthened the relationship with DLW_DEE-S1_ (*R*^2^=0.61, *F*_1,13_=8.6, *P<*0.01; Fig. 1F) and gave the relationship:
(11)

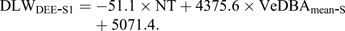
No association was found between DLW_DEE-S2_ and VeDBA_mean-S_ (*R*^2^=0.02, *F*_1,13_=0.3, *P=*0.6; Table 3). As such, the relationship was not further investigated with respect to the influence of additional parameters.

**Table 4.**
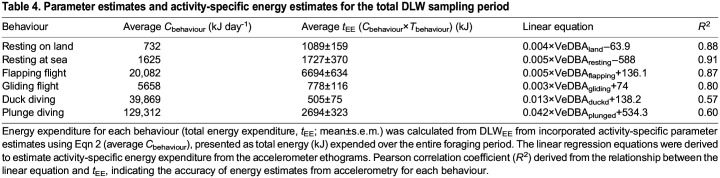
Parameter estimates and activity-specific energy estimates for the total DLW sampling period

## DISCUSSION

The use of accelerometry to determine behavioural patterns has become widespread for birds, mammals and fish (Martiskainen et al., 2009; Whitney et al., 2010; Holland et al., 2009; Bouten et al., 2013; Tsuda et al., 2006; Watanabe et al., 2005) and is increasingly being used for estimating trends in energy expenditure across individuals (Wilson et al., 2006; Green et al., 2009; Halsey et al., 2011, 2008, 2009; Pagano and Williams, 2019). Recently, some *in situ* studies have revealed the importance of species-specific validations over ecologically meaningful durations (Sutton et al., 2021; Pagano and Williams, 2019; Hicks et al., 2020; Elliott et al., 2013; Ste-Marie et al., 2022). In the present study, individuals were sampled on average for >3 days, encompassing a range of natural behaviours. While VeDBA_mean_ was significantly correlated to DLW-derived measures of energy expenditure, models incorporating total distance travelled and time spent at sea during the night were found to provide marginally better correlations for total and at-sea estimates, respectively. These results indicate that accelerometry-derived indices of movement are effective in estimating energy expenditure across this sample of free-ranging Australasian gannets. Furthermore, relationships benefit from the inclusion of additional metrics that are likely to affect energy expenditure, but which may be undetected by accelerometers.

### Metabolic rate and accelerometry-derived indices of energy expenditure

Using the DLW method, the present study estimated the daily energy expenditure of Australasian gannets in early chick rearing to be 3172 kJ day^−1^. This value is approximately 30% greater than previously estimated from *f*_H_ during the same stage of breeding at the same study site (Green et al., 2013) but close to the energy expenditure values from the similarly-sized Cape gannet (*Morus capensis*, 3380 kJ day^−1^) (Adams et al., 1991). While energy expenditure is known to increase during the breeding season in gannets, reflecting the rising demands of offspring provisioning and increasing maintenance costs (Green et al., 2013; Botha and Pistorius, 2018; Gales and Green, 1990; Chappell et al., 1993), the differences observed between the present study and the *f*_H_ estimates are unlikely to be due to the stage of the breeding season.

Alternatively, differences in energy expenditure between the present and previous studies could potentially be attributed to inter-annual variation in foraging conditions. Certain environmental conditions have been shown to impact animal movement and, therefore, may result in differences in energy expenditure (Bryce et al., 2022; Fromant et al., 2022). In the present study, individuals performed twice the number of dives per day compared with the individuals sampled in Green et al. (2013). While there were no data to assess prey availability in either study, changes in marine predator behaviour have been shown to reflect changes in environmental conditions (Hazen et al., 2019). Indeed, strong inter-annual fluctuations in foraging conditions for Australasian gannets at the Pope's Eye colony have previously been observed (Angel et al., 2015). Hence, the higher average energy expenditure observed in the present study could be due to poorer foraging conditions than experienced by individuals in the Green et al. (2013) study.

The distance and duration of a foraging trip have been positively correlated with field metabolic rate (FMR) in central place foraging marine predators (Masden et al., 2010; Ballance et al., 2009). In the present study, total distance travelled and proportion of time spent at sea during the night were important predictors of total and at-sea energy expenditure, respectively. For the total energy expenditure model, the addition of total distance travelled strengthened the relationship with VeDBA; this may be because it reflects the overall amount of movement undertaken. However, for the at-sea period alone, this was not an important predictor.

Rather, the proportion of time spent at sea during the night was important in predicting at-sea relationships. As the energy expenditure of resting on land varies between day and night in gannets (Green et al., 2013), similar patterns may be present in resting at sea behaviour. Indeed, Fauchet et al. (2021) demonstrated that time spent preening was significantly higher during the day. Furthermore, this high-cost behaviour often occurred after dives, probably in response to plumage disturbance. As such, the proportion of time at sea during the night was an important predictor as it may reflect the difference between ‘true’ at-sea resting and active resting, which is interspersed by short bursts of energetically costly behaviours (Thiebault et al., 2014a,b).

In laboratory studies, strong relationships (*R*^2^=0.81–0.91) have been observed between energy expenditure and ODBA*/*VeDBA (Halsey et al., 2009; Wilson et al., 2006; Butler et al., 2004; Enstipp et al., 2011). In comparison, there has been varied success in validating energy expenditure in free-ranging animals. While these studies have demonstrated positive relationships between DLW and accelerometry, others have found no relationship. The weak or unapparent relationships between energy expenditure- and accelerometery-derived estimates may be due to physiological processes (e.g. thermoregulation, digestion) or dive-related costs (e.g. hydrodynamic drag and buoyancy) having a greater influence on energy expenditure than overall dynamic movement (Dalton et al., 2014; Ste-Marie et al., 2022).

In the present study, the strong correlation between accelerometry and DLW demonstrates a clear relationship between movement and energy expenditure in Australasian gannets. The correlations (*R*^2^=0.75–0.86) were analogous to studies that have demonstrated similar accelerometry–DLW relationships (Pagano and Williams, 2019; Jeanniard-du-Dot et al., 2017a; Sutton et al., 2021). The slightly lower correlation observed in *in situ* studies compared with laboratory studies may reflect the influence of inter-individual variability and environmental factors on energy expenditure. The proportion of energy allocated to activity and physiological processes could influence, or indeed mask, relationships between DLW and acceleration. Previous studies have demonstrated that mass is an important factor in energy expenditure relationships, with individuals of smaller body mass expending energy at a higher rate (Costa et al., 1986; Jeanniard-du-Dot et al., 2017a; Sutton et al., 2021; Nagy, 1987). In the present study, body mass along with measurements of tarsus and bill morphometrics were not retained as an important factors in any models. However, during the >3 day study period, multiple foraging trips were conducted and the mass of individuals may have varied as a result of offspring provisioning. If the mass of an individual were to vary dramatically, energy expenditure comparisons may not be valid. For example, a large change in mass between the beginning and end of a foraging trip would make the beginning and end accelerometry and energy expenditure values incomparable. As the present study investigated between-individual (i.e. population) trends, rather that within-individual trends, additional handling times to obtain multiple mass measurements would have been superfluous. Instead, mass measurements were only taken at the beginning and end of the data collection period, revealing no significant differences. This is in line with previous studies suggesting that in relatively large seabirds, mass changes should be negligible over short periods (Weimerskirch et al., 2003). Furthermore, given the small sample size, coupled with the relatively narrow range of values for body mass and each of the measured morphometric variables, it is unsurprising that no relationships were detected between energy expenditure and individual-specific variables in the present study.

### Activity-specific energy expenditure

Variation in the allocation of time towards each behaviour can influence the trade-off between energy expended and acquired (Jeanniard-du-Dot et al., 2017b). Understanding the relationship between energy and specific behaviours allows for inter-individual comparisons to measure the cost of foraging decisions, which have fitness repercussions (Visser and Fiksen, 2013; Collins et al., 2016). The concurrent sampling of accelerometry and DLW in the present study allowed for the calibration of activity-specific energy expenditure estimates across a range of behaviours.

As with previous studies (Elliott et al., 2013; Hicks et al., 2020; Sutton et al., 2021; Jeanniard-du-Dot et al., 2017a), behaviours recorded by accelerometry in the present study were found to incur different energetic costs. The highest energetic costs related to flapping flight and diving behaviours. This was expected given the high level of movement undertaken during these activities. Resting behaviour comprised the greatest proportion of the study period, during which individuals on land have been observed to preen, tail wag and defend their nest and offspring from neighbouring conspecifics. Interestingly, energy expenditure values for at-sea resting were higher than those for on-land resting. This may be due to high-cost preening behaviours occurring within this period (Fauchet et al., 2021). Although it was not possible to quantify preening behaviour in the current study, it has been shown to incur high costs and comprise large proportions of at-sea periods in Australasian gannets (Fauchet et al., 2021) and other seabird species (Croll and McLaren, 1993; McInnes et al., 2017; Thiebault et al., 2014a).

While energy expenditure is influenced by movement, costs of movement can be impacted by environmental conditions (Bryce et al., 2022). For example, behaviours such as flapping flight may be influenced by wind speed and direction (Amélineau et al., 2014). However, as individuals were sampled over the same time period, it is likely that they experienced similar meso-scale environmental conditions (i.e. temperature, wind speed). This may have resulted in strong correlations being observed for resting and flying behaviours.

Energy expended during diving should be affected by dive characteristics such as duration, depth and underwater movements (Ropert-Coudert et al., 2009), all of which may have varied considerably between individuals for both plunge diving and surface diving. Although plunge diving in gannets involves a high acceleration rate (up to 7 ***g***) (Yang et al., 2012) and is thought to be of lower energetic cost because of the effect of gravity (Green et al., 2010), the present study suggests that it is the most energetically expensive behaviour. From an optimal foraging perspective, while diving represents the greatest opportunity for energy gain, it should also be the least performed because of its associated energetic costs (Acevedo-Gutiérrez et al., 2002). Correspondingly, moderate correlations were detected between energy expenditure and activity-specific VeDBA for both plunge- and duck-diving behaviours. The greater variation in energy expenditure during diving periods may be related to variation in the intensity of dive behaviour in response to prey characteristics. Such variation could potentially explain the lower correlations with VeDBA derived from the activity-specific approach observed in both plunge- and duck-diving events.

Of the two at-sea estimates of energy expenditure, the method utilising activity budgets to determine on-land energy expenditure (i.e. DLW_DEE-S1_) performed better than the method using published values (i.e. DLW_DEE-S2_). The results of the present study demonstrated that resting behaviour, both on land and at sea, is an important component of daily energy expenditure. While DLW_DEE-S1_ performed reasonably well as a result of the ability to account for land costs using activity-specific equations, the on-land energy constants from published values used to calculated DLW_DEE-S2_ were not able to account for inter-individual variation in on-land energy expenditure. The vast difference in success of the two methods highlights the importance of validating indices of energy expenditure and determining the best practices in estimating energy expenditure. This is especially important considering the use of published constants is commonplace (Collins et al., 2016; Ladds et al., 2018; Tatler et al., 2021). The present study demonstrates that non-invasive proxies of energy expenditure derived from accelerometry may be more accurate than the use of published constants to determine estimate energy expenditure.

South-eastern Australia is a region of rapid oceanic warming, with anticipated changes to the availability of pelagic fish likely to cause cascading impacts (Crossin et al., 2014; Lough and Hobday, 2011). Therefore, it is important to understand how animals in this region interact with their environment and how their energy expenditure may vary under changing conditions. Energy expenditure calculated from activity budgets proved to correlate well with actual energy expenditure. As such behaviours have been shown to vary in relation to local prey availability in Australasian gannets (Angel et al., 2015), the results of the present study indicate accelerometry can also be used to investigate how individuals alter their energy budgets in response to environmental variability. The calculated activity-specific coefficients for VeDBA may be useful to apply to future studies when investigating time–activity budgets.

The reason that the at-sea relationships performed poorly in comparison to relationships encompassing the total sampling period may be that the processing of the DLW data generated greater error. For example, if a variable has 7.5% error associated and half is subtracted, the effective error increases. Indeed, in the present study, where animals spent almost 50% of their time at the colony, the *R*^2^ values for the at-sea relationship (0.42) was approximately half that observed between DLW and VeDBA_mean_ of the total sampling period. It is likely that the comparison between the two proxies, both with associated error (Speakman, 1997), resulted in a reduced predictive ability of the at-sea relationships. This should be considered when attempting to estimate energy expenditure over shorter durations (i.e. single foraging trips).

In summary, the results of the present study have shown that accelerometry provides a relatively simple non-invasive method for estimating energy expenditure in Australasian gannets. The addition of the movement metrics distance travelled and proportion of time at sea during the night increased the predictive power of the total and at-sea models, respectively. While species-specific validations are still needed, this study provides further evidence that accelerometry is an accurate proxy of energy expenditure in seabirds. Expanding on previous studies of shorter duration, the results of the present study show that the relationship between DLW and accelerometry is maintained over extended periods. Hence, accelerometers may provide a relatively inexpensive tool for enabling large numbers of individuals to be sampled over ecologically meaningful time scales.

## Supplementary Material

10.1242/jexbio.246922_sup1 Supplementary information

Table S1. Data used in the present study
