# Supplementary material for: Determining energy expenditure in a large seabird using accelerometry
Source: J Exp Biol. 2023 Dec 6;226(23):jeb246922. doi: 10.1242/jeb.246922 (PMC10714144; doi:10.1242/jeb.246922)
Supplement: Supplementary information [file jexbio-226-246922-s1.pdf]

**Table S1. Data used in the present study**

Available for download at
https://journals.biologists.com/jeb/article-lookup/doi/10.1242/jeb.246922#supplementary-data

Journal of Experimental Biology • Supplementary information
